# Torture exposure and the functional brain: investigating disruptions to intrinsic network connectivity using resting state fMRI

**DOI:** 10.1038/s41398-022-01795-3

**Published:** 2022-01-26

**Authors:** Belinda J. Liddell, Pritha Das, Gin S. Malhi, Kim L. Felmingham, Tim Outhred, Jessica Cheung, Miriam Den, Angela Nickerson, Mirjana Askovic, Jorge Aroche, Mariano Coello, Richard A. Bryant

**Affiliations:** 1 School of Psychology, UNSW Sydney, Sydney, NSW 2052 Australia; 2 Department of Psychiatry, Faculty of Medicine and Health, Northern Clinical School, The University of Sydney, Sydney, NSW Australia; 3 Academic Department of Psychiatry, Royal North Shore Hospital, Northern Sydney Local Health District, St Leonards, Sydney, NSW 2065 Australia; 4 CADE Clinic, Royal North Shore Hospital, Northern Sydney Local Health District, St Leonards, Sydney, NSW 2065 Australia; 5 School of Psychological Sciences, University of Melbourne, Parkville, VIC 3010 Australia; 6 NSW Service for the Treatment and Rehabilitation of Torture and Trauma Survivors (STARTTS), Carramar, NSW 2163 Australia

**Keywords:** Biomarkers, Psychology

## Abstract

Torture has profound psychological and physiological consequences for survivors. While some brain structures and functions appear altered in torture survivors, it is unclear how torture exposure influences functional connectivity within and between core intrinsic brain networks. In this study, 37 torture survivors (TS) and 62 non-torture survivors (NTS) participated in a resting-state fMRI scan. Data-driven independent components analysis identified active intrinsic networks. Group differences in functional connectivity in the default mode network (DMN), salience network (SN) and central executive network (CEN) of the triple network model, as well any prefrontal network, were examined while controlling for PTSD symptoms and exposure to other potentially traumatic events. The analysis identified 25 networks; eight comprised our networks of interest. Within-network group differences were observed in the left CEN (lCEN), where the TS group showed less spectral power in the low-frequency band. Differential internetwork dynamic connectivity patterns were observed, where the TS group showed stronger positive coupling between the lCEN and anterior dorsomedial and ventromedial DMN, and stronger negative coupling between a lateral frontal network and the lCEN and anterior dorsomedial DMN (when contrasted with the NTS group). Group differences were not attributed to torture severity or dissociative symptoms. Torture survivors showed disrupted dynamic functional connectivity between a laterally-aligned lCEN that serves top-down control functions over external processes and the midline DMN that underpins internal self-referential processes, which may be an adaptive response to mitigate the worst effects of the torture experience. This study provides a critical step in mapping the neural signature of torture exposure to guide treatment development and selection.

## Introduction

Torture, according to the United Nations Convention Against Torture and Other Cruel, Inhuman or Degrading Treatment or Punishment (UNCAT), is defined as an intentional act of physical or psychological harm inflicted on a person for the purposes of obtaining information, punishment, intimidation and/or discrimination of any kind by a person in or acting for a person in an official capacity [1]. It is most commonly characterised by exposure to interpersonally harmful events delivered in captivity with the purpose of evoking a complete loss of control [2]. Not only does torture evoke extreme emotions like fear and shame, but the insidious intent behind acts of torture also targets the whole person, undermining their self-concept, social bonds and moral frameworks [3–5]. Torture is expressly prohibited by international law [6] and yet remarkably it continues to be practiced unabated in 141 countries worldwide [7]. As a consequence, a staggering one in five conflict-affected people and refugees are estimated to be survivors of torture [8]. While torture is commonly experienced in the context of other potentially traumatic events (PTEs) related to war, conflict or persecution, the specific adverse and often chronic physical, psychological and social effects of torture on survivors have been well documented [9]. Not surprisingly, torture exposure is the strongest predictor of posttraumatic stress disorder (PTSD) in refugees and conflict-affected people globally [8], and survivors can experience long-lasting psychological sequelae that includes chronic and severe psychopathology [10, 11], dissociative reactions and disturbances to self-identity [12, 13], difficulties regulating emotions [5, 14], cognitive symptoms—e.g. memory and concentration impairments [15] and marked social difficulties—e.g. attachment disruptions, interpersonal problems [16, 17]. In terms of post-torture treatment, it is likely that the complexity of torture experiences and its aftermath contributes to relatively poorer responses to first-line interventions for PTSD [18]. Understanding the specific effects of torture exposure itself on the brain and its functional networks, regardless of the range of psychological ramifications, could be an important first step for identifying core mechanisms that could be new treatment targets for survivors [19].

It has been proposed that the unique characteristics of the torture experience could affect the functional architecture of the brain [16, 20], but research into torture’s direct effects on core brain networks has been relatively limited. Research to date indicates that torture affects the composition and functionality of brain structures. For example, studies have reported volumetric changes [21, 22], cortical thinning [23] and white matter connectivity differences [21] in torture survivors with PTSD compared to healthy controls, with the degree of structural differences being associated with trauma exposure [22] or depression symptoms [23]. Functional studies have shown that torture survivors with PTSD differentially engage threat-related, emotion regulation [24–26] or sensory processing [27, 28] brain systems compared to either torture survivors without PTSD or healthy controls [16]. The prefrontal cortex may be particularly affected by torture exposure, both structurally [22, 23] and functionally [24, 26]. While these studies have afforded some insights into the neural effects of torture, the derivation of robust conclusions has been difficult because of methodological variation between studies [16], which include: (1) the nature of participant groupings, which have lacked a specific focus on torture exposure itself by either including participants with exposure to multiple conflict-related events including torture in the one group [22, 24, 25, 29], not distinguishing between torture exposure and PTSD diagnosis [21, 22], or else comparing torture survivors to healthy controls without torture histories [22, 24], which may exaggerate the specific effects of torture [16]; (2) the focus on fMRI and MEG studies with task-related designs, which precludes insights into how torture affects the functional dynamics of brain networks.

In recent years a growing number of researchers have attempted to understand the dynamics of the brain at rest using a network approach [30], in which networks represent functional modules of the brain that provide valuable diagnostic and prognostic information [31]. The triple network model, comprising the default mode network (DMN), salience network (SN) and central executive network (CEN), has been proposed to understand brain disruptions in cognitive and affective disorders [32], including PTSD [33]. In healthy functioning, the SN detects, filters and integrates arousing or novel information, the CEN directs attention and cognitive control resources towards goal-directed and external-based information [34] and the DMN enables self-referential processes and integrates behavioural responses with internal processes [35, 36]. These three networks work in operational balance to facilitate metacognitive functions and integrate external and internal processes [37, 38]. We hypothesise that brain disturbances following torture exposure may involve these three critical networks.

Differential connectivity patterns within and between these brain networks have been observed in PTSD, which is marked by a hyperactive SN and reduced CEN-DMN connectivity [33]. While task-based brain imaging studies indicate that PTE exposure may modulate the functional brain in meaningful ways [39, 40], the majority of resting-state fMRI (rsfMRI) studies have examined the role of PTSD symptoms, and only a few have considered the specific effects of trauma exposure itself irrespective of PTSD. For example, studies that examined the effects of repeated trauma exposure observed altered functional connectivity between the SN and DMN in firefighters [41], and DMN and visual networks in earthquake survivors [42]. In a study of survivors of intimate partner violence, comparisons between those with and without PTSD showed no differences in intrinsic connectivity patterns 3 weeks or 6 months following assault [43]. Findings from these studies are mixed but suggest that trauma exposure itself may affect intrinsic brain networks [39], but no study to date has specifically focused on torture trauma. Understanding how torture affects the dynamic connectivity of these key functional networks is important in terms of establishing the core neural effects of torture exposure.

In this study, we examined how torture exposure modulates functional connectivity within and between the three networks of the triple network model comprising the DMN, SN and CEN, as well as any prefrontal cortical network due to prior evidence that its functioning is affected by torture [24–26]. To overcome previously identified methodological issues and to focus directly on the effects of torture exposure, we recruited a sample of torture survivors with a refugee background as the target group, and a comparison group comprising refugees without a history of torture exposure, while statistically controlling for PTSD symptoms and exposure to non-torture PTEs. Furthermore, we used a resting-state fMRI paradigm, which is a reliable method for overcoming problems with task-related fMRI with smaller sample sizes, and for elucidating how large-scale intrinsic brain networks connect and interact [19]. Using a data-driven approach to identify active networks, we examined both within-network and between-network connectivity to provide a comprehensive picture on how torture exposure affected intrinsic network connectivity patterns. First, we investigated torture vs non-torture survivor group differences in the functional connectivity within identified networks as measured using spatial maps (SM) and spectral power of time series (SP) [44]. We then examined group differences in dynamic functional network connectivity (dFNC)—a highly powerful and replicable tool for mapping network integration patterns [45]—that is, the strength of between network communication over time. Our exploratory hypothesis was that torture survivors (TS group) would demonstrate differential functional connectivity within and between the DMN, CEN, SN and prefrontal networks compared to non-torture survivors (NTS group).

## Methods

### Participants

Participants were 104 individuals with a refugee background settled in Australia. All participants had conflict and displacement-related trauma histories, with 39 participants reporting personal experience of torture. Recruitment primarily occurred via a torture and trauma treatment service in Sydney Australia (NSW Service for the Treatment and Rehabilitation of Torture and Trauma Survivors- STARTTS), supplemented by recruitment via other refugee and community services. Inclusion criteria were determined via pre-screening and included being over 18 years old, no history of psychosis, bipolar, alcohol-use, substance-use or neurological disorder, no moderate-severe traumatic brain injury, no current suicidality and able to meet MRI safety criteria (no metallic implants or injuries, irremovable piercings, etc.). All participants who met inclusion criteria provided written informed consent in their preferred language as approved by the Northern Sydney Local Health District Human Research Ethics Committee (NSLHD HREC 1210-342M). Participants were reimbursed for participating in the study and were provided with return transport from the MRI scanning session.

### Defining torture exposure

Torture exposure was specified according to United Nations Convention Against Torture (UNCAT) definition: an act of extreme physical or psychological pain and suffering inflicted on a person for the purposes of obtaining information, punishment or intimidation by an official entity [1]. We verified torture exposure with participants in three steps: (1) endorsement of the torture item in the Harvard Trauma Questionnaire (HTQ) [46]; (2) completion of a torture-experiences questionnaire based on a previous measure [10] but developed for this study to index the degree, type and extent of torture exposure; and (3) completion of the Torture Survivor Check-List (TSCL) [47] to verify whether events reported met the UNCAT definition. We computed a torture severity index, comprising count of exposure to different torture event types and duration of this exposure (modelled on [25]; see Supplementary Material Table 1 for the description of torture event types).

### Diagnostic and symptom measures

Participants completed a clinical interview with a psychologist and a professional interpreter if required. The interview elicited demographics, displacement, torture and trauma histories and an assessment of selected mental health symptoms. Trauma exposure was assessed by the HTQ [46], which indexes exposure to 16 potentially traumatic events (PTEs) common to refugees, including torture. We used the PTSD Symptom Scale-Interview (PSS-I) to assess PTSD symptoms experienced within the last 2 weeks according to DSM-5 criteria [48]. A total score was computed to determine PTSD symptom severity (internal consistency was strong; Cronbach α = 0.93), and PTSD diagnosis was computed by an algorithm according to DSM-5 criteria, with scores of 2 or above (at least somewhat or 2–4 times per week) indicating the presence of each symptom.

### MRI data acquisition

MRI scanning was completed within 1–2 weeks following the clinical interview, using a 3T Siemens Magnetom Trio Scanner based at the Advanced Research and Clinical High-field Imaging (ARCHI) facility in Sydney. A T2*-weighted gradient-echo echo-planar imaging (EPI) sequence (29 axial slices, slice thickness 4 mm with 1 mm gap, repeat time (TR) = 2000 ms, echo time (TE) = 35 ms, flip angle (FA) = 70^o^, 64 × 64 matrix, FOV = 240 mm, in-plane resolution = 3.75 mm) was used to acquire 155 whole-brain volumes of functional data. This volume of data has previously been shown to robustly capture different connectivity states [45, 49, 50]. Subjects were instructed to keep their eyes open during the functional scan and stare passively at a foveally presented fixation cross in order to facilitate network delineation (as opposed to an eyes-closed resting state) [51] and to prevent participants from falling asleep. Head motion during scanning was limited by using foam pads inserted on each side of the participant’s head. All participants were awake and alert at the start and conclusion of scanning.

### fMRI data analysis

#### Pre-processing of fMRI data and identification of components using spatial ICA

Pre-processing was done using a combination of toolboxes – SPM8 (https://www.fil.ion.ucl.ac.uk/spm/software/spm8/) and GIFT (https://trendscenter.org/software/gift/). Group spatial ICA [52, 53] as implemented in the GIFT software was used to identify components from resting-state data using the minimum description length (MDL) criteria [54] (refer to Supplementary Material for complete analysis information).

#### Selection of networks of interest and post-processing to remove remaining noise

Networks for analysis were chosen on the basis of three conditions. First, a component’s peak activation cluster should fall on grey matter and it should show low spatial overlap with known vascular, ventricular, susceptibility, and edge regions corresponding to head motion. Second, a component should show more spectral power in the low-frequency range (0.01– 0.10 Hz) compared to the high-frequency range (0.15–0.25 Hz) [44]. Finally, in line with our hypotheses, a component should represent either DMN, CEN, SN or any frontal networks showing primary activity in the prefrontal cortices. For details on the removal of remaining noise, see Supplementary Materials.

#### Within and between network functional connectivity

The MANCOVAN toolbox within GIFT software was used to determine within-network functional connectivity. Within-network connectivity was measured using (1) the intensity of each network spatial map (SM), which represents the connectivity and degree of co-activation within a network; and (2) the distribution of spectral powers (SP) across the time course at different frequencies, which represents the level of coherent activity within a network. To determine functional connectivity between networks, the temporal dynamic functional network connectivity (dFNC) toolbox within the GIFT software was used. Data analyses using dFNC have been conducted in keeping with procedures outlined in previous studies [50, 55]; for details refer to Supplementary Material.

#### Between group differences within-network and between network functional connectivity

Differences between groups (TS vs NTS) were investigated using two-sample *t*-tests after controlling for age, total PTSD severity and past trauma count (apart from torture exposure)—variables which significantly differed between groups (see Results, Participant Characteristics). We controlled for PTSD symptoms as the aim of this study was to examine the specific effects of torture exposure on functional network connectivity. We first considered group differences in a spatial map and spectral power outputs, representing differences in connectivity strength within networks. We then considered group differences in dFNC, reflecting differences in network connectivity patterns between groups. Results were corrected for multiple comparisons using the false discovery rate correction (*p* < 0.05 FDR-corrected). For dFNC, this was done for each of the four states, also *p* < 0.05 (FDR-corrected).

## Results

### Participant characteristics

After removing five participants due to the excess movement (extreme motion defined as shifts in the translation of >1 voxel size (3.75 mm) or rotation >2°), our final sample consisted of 99 participants of which *N* = 37 were survivors of torture (TS group) and *N* = 62 were non-torture survivors (NTS group). Demographic and symptom variables are presented in Table 1, with a Bonferroni-corrected threshold of *p* < 0.004 applied to group comparisons. The TS group was older (*t*(97) = 3.08, *p* = 0.003) and more likely to be male (χ^2^(1) = 12.33, *p* < 0.001) than the NTS group. There were no significant group differences in terms of marital, employment or visa status, education, country-of-origin, time in Australia, psychotropic medication, or current psychological treatment (see Table 1). The rates of PTSD diagnosis were also equal between the groups (χ^2^(1) = 1.01, *p* = 0.32), but the TS group reported more severe PTSD symptoms at an uncorrected threshold (*t*(97) = 2.15, *p* = 0.034), greater exposure to different PTE types (excluding torture) (*t*(97) = 3.17, *p* = 0.002). We, therefore, elected to control for age, non-torture trauma exposure and PTSD symptoms in fMRI analyses, given that these factors may influence findings and that we were specifically interested in the effects of torture exposure on network activity and connectivity.

**Table 1:** Participant demographic and mental health symptoms.

		Torture survivor (TS) group (*N* = 37)		Non-torture survivor (NTS) group (*N* = 62)		Group difference *p* value
		*N* / mean	% / SD	*N* / mean	% / SD	
Age (years)		42.11	11.82	35.11	10.37	*p* = 0.003*
Sex	Male	32	86.5%	32	51.6%	*p* < 0.001*
	Female	5	13.5%	30	48.4%	
Marital status	Married	24	64.9%	30	48.4%	*p* = 0.007
	Widow/widower	4	10.8%	0	0%	
	Divorced/separated	1	2.7%	5	8.1%	
	Single/never married	8	21.6%	27	43.5%	
Education	No education	2	5.6%	2	3.2%	*p* = 0.851
	Completed primary school	8	22.2%	13	21.0%	
	Completed high school	9	25.0%	20	32.3%	
	Completed tertiary or vocational training	17	47.2%	27	43.5%	
Employment	Employed (Full or part-time)	8	21.6%	10	16.1%	*p* = 0.445
	Studying	5	13.5%	15	24.2%	
	Unemployed	5	13.5%	7	11.3%	
	Unable to work	17	45.9%	22	35.5%	
	Home duties or retired	2	5.4%	8	12.9%	
Country-of-origin	Iran	14	37.8%	25	40.3%	*p* = 0.223
	Iraq	4	10.8%	16	25.8%	
	Sri Lanka	2	5.4%	2	3.2%	
	Other^#^	17	45.9%	20	30.6%	
Visa status	Secure visa	17	45.9%	35	56.5%	*p* = 0.311
	Insecure visa	20	54.1%	27	43.5%	
Medication	Psychotropic medication	9	24.3%	10	16.1%	*p* = 0.316
Treatment	Psychological treatment	16	55.2%	27	49.1%	*p* = 0.596
Time in Australia (years)		5.74	8.23	3.31	4.31	*p* = 0.100
PTSD diagnosis (DSM-5)		15	40.5%	19	30.6%	*p* = 0.316
PTSD Symptom severity (PSS-I; sum)		26.59	14.46	20.21	14.16	*p* = 0.034
PTE exposure (HTQ); excluding torture item (count)		11.70	3.77	9.53	2.98	*p* = 0.002*
Dissociative symptoms (DES-T; sum); see posthoc analyses.		124.72	114.75	74.68	89.50	*p* = 0.022

Two-sample *t*-tests were performed for continuous variables and Chi-square group tests for categorical variables.

*p < 0.004 (Bonferroni-corrected).

^#^Other countries of origin include Afghanistan, Bosnia-Herzegovnia, Cambodia, Bhutan, Morocco, Myanmar, Chile, Fiji, Ghana, Kuwait, Laos, Nigeria, Tibet and Vietnam.

Torture survivors were subject to a wide range of torture-related events, including sexual torture (24.3%), physical torture (81.1%) and psychological torture (78.4%), with the majority of participants experiencing events over a prolonged period of time (Supplementary Material Table 1). The mean torture severity score was 10.9 (SD = 5.2, range 1–19), mean total duration of exposure to torture-related events was 1.22 years (SD = 1.81, range 0.1–6.05 years), and mean time since torture exposure was 10.53 years (SD = 8.63, range 2–30 years).

### Network identification

**Fig. 1:**
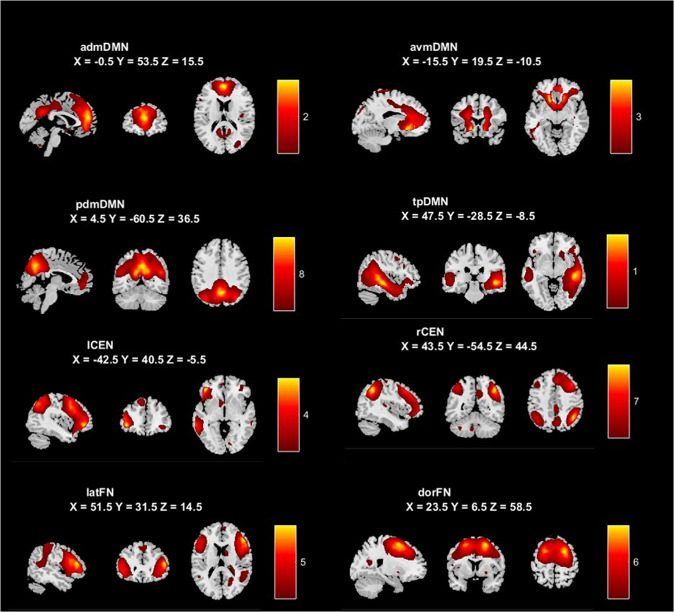
Eight chosen networks of interest from ICA analysis. MNI coordinates are provided for the slices presented. admDMN anterior dorsomedial default mode network, avmDMN anterior ventromedial default mode network, pdmDMN posterior dorsomedial default mode network, tpDMN temporoparietal default mode network, lCEN left central executive network, rCEN right central executive network, latFN lateral frontal network, dorFN dorsal frontal network.

From a total of 25 networks (see Supplementary Material, Fig. 1), determined using MDL criteria [54], eight were chosen as our networks of interest according to our selection criteria outlined in the Methods. The spatial maps for each network are presented in Fig. 1, with the primary regions within each network provided in Supplementary Material Table 2. Four components represented spatial divisions of the default mode network (DMN): (1) anterior dorsomedial default mode network (admDMN) spanning dorsal components of the bilateral medial frontal gyrus, superior frontal gyrus and anterior cingulate cortex (ACC), as well as incorporating clusters in the precuneus and posterior cingulate cortex (PCC); (2) anterior ventromedial default mode network (avmDMN) spanning bilateral ventral regions of the ACC and prefrontal gyri; (3) posterior dorsomedial default mode network (pdmDMN) focused on bilateral precuneus, posterior cingulate, and dorsal temporoparietal regions; (4) temporoparietal default mode network (tpDMN) comprising bilateral ventral temporoparietal and occipital regions, as well as the insula. Two components represented the central executive network (CEN): the left (lCEN) and right central executive network (rCEN) spanning lateralized lateral prefrontal and parietal regions. A further two components represented prefrontal networks: dorsal (dorFN) and lateral (latFN) frontal networks spanning bilateral dorsal and ventral regions of the frontal lobe respectively. The cluster stability and quality of the chosen networks were very high (Iq > 0.9). No components representing the salience network were identified in this sample.

### Effects of torture exposure on within-network connectivity

**Fig. 2:**
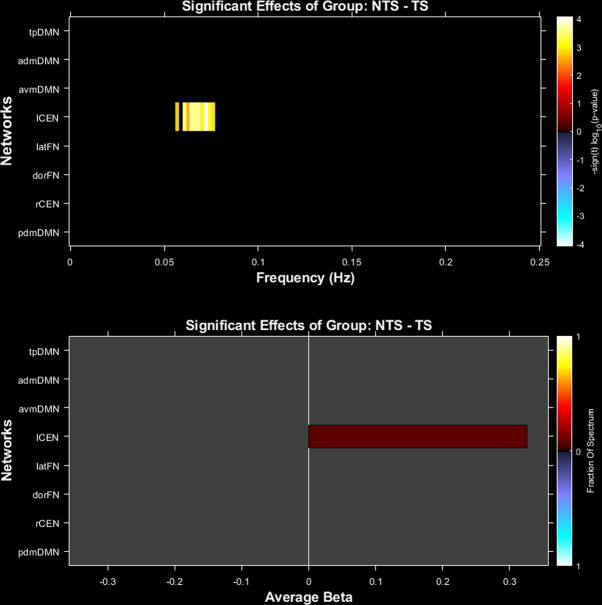
Group differences in spectral power. Differences between torture survivor (TS) compared to non-torture survivor (NTS) groups in spectral power in the left central executive network (lCEN). Effect size is also provided.

Only one network from the eight selected networks demonstrated differences between torture-exposure groups—namely in the lCEN. The TS group displayed *less* spectral power in the low-frequency range (0.05–0.1 Hz) in the lCEN compared to the NTS group (*p* < 0.05, FDR-corrected); see Fig. 2, while controlling for age, PTSD symptoms and non-torture PTE exposure.

### Effects of torture exposure on *between* network connectivity

**Fig. 3:**
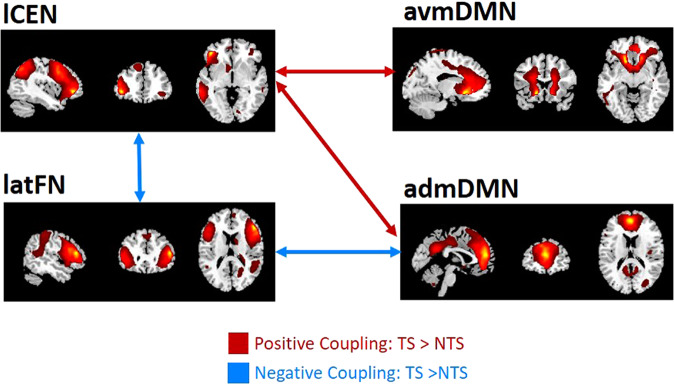
Group differences in between-network connectivity patterns. The torture group showed increased positive functional connectivity between the lCEN and admDMN and avmDMN compared to the NTS group (represented by red arrows), and greater negative functional connectivity between the lCEN and latFN and latFN and admDMN (represented by blue arrows) (*p* < 0.05 FDR-corrected).

We assessed torture group differences in between network connectivity using dynamic functional network connectivity (dFNC). Of a possible 28 between network connections, four were significant after correcting for multiple comparisons (*p* < 0.05 FDR-corrected); see Fig. 3; means and SDs are presented in Supplementary Material Table 3.

Two significant dFNCs between groups featured stronger positive functional connectivity between the CEN and anterior divisions of the DMN in the TS group. Specifically, we observed increased *positive coupling* between the lCEN and admDMN and the lCEN and avmDMN in the TS compared to the NTS group. Significant differences were also observed in the dynamic functional connectivity of the latFN. Specifically, in comparison to the NTS group, the TS group showed stronger *negative coupling* between the latFN and two other networks namely, the lCEN and the admDMN (Fig. 3).

### Secondary analyses: Correlations with dissociative symptoms and torture severity

The pattern that emerged for the TS group of stronger positive coupling between the lCEN and DMN resembles connectivity profiles reported for the dissociative subtype of PTSD [56, 57]. Therefore, we conducted a secondary analysis to test whether our dFNC findings could be attributed to the fact that dissociative symptoms were higher in the TS (vs NTS) group. Dissociative symptoms were assessed in our study using the Dissociative Experiences Scale Taxon (DES-T) [58], an eight-item measure derived from the original DES-II [59] that specifically considers pathological dissociative symptoms —particularly amnesic dissociation, depersonalisation and derealization [58]. Internal consistency was satisfactory (Cronbach α = 0.71). The TS group reported higher levels of dissociative symptoms (*t*(91) = 2.33, *p* = 0.022) compared to the NTS group. In a series of bivariate correlations with extracted time-series data (significance threshold *p* < 0.0125 Bonferroni-corrected), we observed a trend whereby dissociative symptoms were positively correlated with latFN-lCEN coupling (*r* = 0.183, *p* = 0.08), but no significant correlations were observed. Considering just the torture group, this same correlation between dissociative symptoms and latFN-lCEN connectivity was not significant (*r* = 0.280, *p* = 0.115). These findings do not support the notion that differences in dynamic functional connectivity observed between the groups were attributed to higher dissociative symptoms. We also examined whether dFNC patterns were related to torture experiences in the TS group, but no significant correlation was observed between the dFNC time-series and torture severity index (*p* < 0.05).

## Discussion

This study investigated the effect of exposure to torture on functional brain networks and found differential intrinsic network connectivity in a group of torture survivors (TS) compared to non-torture survivors (NTS). Torture exposure appears to be primarily associated with differences in the functioning of the left CEN (lCEN). We observed alterations in lCEN activity within-network, as well as the dynamic connectivity between lCEN and the DMN in the TS group relative to the NTS group. Specifically, we found greater positive coupling between the lCEN and the midline anterior medial (admDMN) and ventral DMN (avmDMN) in the TS group. The TS group also demonstrated more negative coupling between the lateral frontal network (latFN) and the lCEN and anterior dorsomedial DMN (admDMN), compared to the NTS group. Torture group differences were observed irrespective of the impact of PTSD symptom severity and exposure to other PTE types such as conflict or displacement trauma and were not attributable to dissociative symptoms nor torture severity. Considering mechanisms, our novel findings suggest that torture exposure is associated with specific changes in the left CEN that may drive a dynamic functional imbalance between the lateral (CEN) and medial (DMN) regions of the brain. The functional consequences of these dynamic functional connectivity differences for torture survivors are likely to be clinically relevant and may include significant difficulties in self-regulation following torture exposure that persist over the long-term.

Our study found that the key impact of torture on resting state network activity lay within the left CEN, where we observed less spectral power in the low-frequency band in the TS group. The signal from lower frequency bands is associated with the integration of large-scale neural networks and long-distance connectivity. Our finding of reduced spectral power in the lCEN suggests that not only long-distance connectivity among brain regions within this network could be disrupted in the TS group but that also, integration between large-scale networks be also affected. This is supported by our findings of impaired lCEN functional connectivity with other cortical networks (admDMN, avmDMN and latFN) in the TS group (relative to NTS group). Moreover, differences in this frequency range also suggests that metabolic processes may be disrupted in the TS group [60]. These findings complement existing task-related and structural MRI studies, although we note that this is the first study to observe resting-state network functioning differences in torture survivors. For example, a task-related fMRI study observed stronger lateral prefrontal cortical (part of the CEN)—hippocampus coupling during threat face processing in male torture survivors compared to non-torture survivors, as moderated by level of non-torture PTE exposure [26]. Other structural brain studies focusing on childhood maltreatment have demonstrated that dorsolateral prefrontal cortex volume diminishes following trauma [61]—a finding that is consistent with studies in torture survivors that have demonstrated grey matter volume reductions across the brain including the prefrontal cortex [22, 23]. As we did not examine structural differences in this study, we cannot attribute lCEN functional alterations to structural changes, but at present, this study clearly points to lCEN functional changes in the TS group and perturbations in coupling with medial networks.

Our findings suggest that torture exposure is associated with alterations in the communication between lateral CEN and medial DMN networks, where we observed greater positive (lCEN – admDMN/avmDMN) and negative (latFN – admDMN/lCEN) between network coupling in the TS, compared to NTS, group. Previous studies have demonstrated that the anterior PFC is functionally heterogenous and underpins multiple metacognitive processes [38]. While the lateral PFC (i.e. a key region in the CEN) is critically involved in information processing [38] and exerts top-down executive control over emotional processing [37], the medial PFC (i.e. DMN) subserves self-referential, social and emotional internal processes [35]. Connectivity between the lateral-medial PFC, therefore, appears vital for integrating external-internal processes [37]. Our observations of stronger positive coupling between lCEN- anterior DMN in the TS compared to the NTS group could reflect a functional change in the communication between these lateral-medial PFC systems.

CEN-DMN hypercoupling has also been observed in the dissociative subtype of PTSD [56, 57], which appears more common amongst survivors of extreme chronic trauma like childhood maltreatment [56, 62, 63]. It has been suggested that increased CEN-DMN coupling in dissociative PTSD could reflect the increased reliance on internal problem-solving processes in response to a stressful external environment [56]. While this mechanism may also have relevance for understanding the neural impact of torture, our secondary analyses suggest that even though torture survivors reported higher dissociative symptoms in our study, we did not observe a strong relationship between dissociative symptoms and brain connectivity patterns. This suggests that torture may independently shape hyperconnective CEN-DMN, regardless of dissociative symptoms, in our sample. It is therefore possible that dissociative symptoms in torture survivors may be reflected in a unique network connectivity mechanism that does not involve CEN-DMN hyperconnectivity. However, given we used a data-driven approach to identify active networks,it is possible there are components of these networks that simply were not active in this sample that might correlate with dissociative symptoms. Future studies could focus on the role of dissociative symptoms to resolve some of these questions.

Our findings reflect models of torture that posit ‘shut-down’ behaviours, including dissociative, withdrawal or emotional numbing symptoms, serve an adaptative function when exposed to torture [13]. Such responses may assist in suppressing or ‘over-regulating’ emotional reactivity, inhibiting difficult memories and managing psychological symptoms to ensure immediate coping and survival, but may be less adaptive in the longer term after the cessation of torture-related events. This is consistent with the fact that we did not identify a salience network active in our study. Our findings highlight a possible brain network-based mechanism behind this over-regulation response - that is, increased CEN-DMN dynamic functional connectivity, with possible consequences including emotional rigidity, inflexibility, withdrawal, problems with self-regulation and difficulties responding to positive interpersonal situations [62], all of which have been observed in torture survivors [26]. Torture exposure itself may therefore fundamentally alter the way intrinsic networks of the brain—in particular the CEN and DMN—function and connect. From a clinical perspective, this means that despite the range of complex and long-term psychological responses to torture events [5, 10–17], clinical interventions might include core components that target dysregulated CEN-DMN functional connectivity. This aligns with new calls to utilise resting-state fMRI findings to understand the heterogeneity of the post-trauma response to identify more homogenous subtypes [19], which could include brain mechanisms disrupted by exposure to specific traumatic events like torture. Proper evaluation of how the diverse psychological sequelae of torture may impact intrinsic neural networks requires the study of large samples of torture survivors, together with a detailed examination of their different psychological profiles.

### Limitations

There are a number of potential limitations of this study to note when considering the findings. Our sample size is comparable to similar resting-state studies conducted in traumatised populations [41], however, only 37 participants with a torture history participated here and hence, our findings will necessitate replication in an independent and larger sample. Our sample size precluded examining PTSD diagnosis, and as such, the role of PTSD symptomatology or diagnosis could be the focus of future studies. We also cannot rule out the contribution of other psychological symptoms, including depression, which could also be considered in future investigations. Further, the torture survivor group were mostly male. We did not control for sex differences in our analysis, as a predominantly male sample is consistent with the fact that globally, torture survivors are more likely to be male [9, 21, 22]. Future studies should focus on recruiting female torture survivors to examine its role in brain networks. Our sample included some participants currently receiving psychological or psychotropic medication treatments, which may have affected findings. However, treatments were stable at the time of fMRI testing for at least 6 weeks prior, which may have minimised the effect of treatment on network activity, and moreover, no group differences were observed. Based on theoretical and empirical evidence, we focused on the three networks of the triple network model and prefrontal networks in our analysis, but there may be other networks that we did not consider that may also play an important role. This study is also cross-sectional, and hence, we cannot be certain about the causal role of torture in explaining the network differences we observed.

## Conclusions

Torture is a significant human rights violation that has long-lasting physical and psychological consequences for survivors. This study found that torture exposure affected functional connectivity within and between core intrinsic brain networks, most prominently within the left CEN and stronger positive coupling with medial dorsal and ventral divisions of the DMN, and stronger negative coupling between the lateral frontal and admDMN/lCEN. Mapping of disrupted connectivity between these identified networks that underpin vital metacognitive and self-referential emotional processes provides critical insights into the long-term impact of torture on functional brain systems.

## Supplementary information

Supplementary Material for paper

## Data Availability

The data included in this study are not publicly available because of the sensitivity of the data collected and the risk that sharing it could violate the participants’ confidentiality.
