# Correction: Torture exposure and the functional brain: investigating disruptions to intrinsic network connectivity using resting state fMRI

**DOI:** 10.1038/s41398-022-01839-8

**Published:** 2022-02-21

**Authors:** Belinda J. Liddell, Pritha Das, Gin S. Malhi, Kim L. Felmingham, Tim Outhred, Jessica Cheung, Miriam Den, Angela Nickerson, Mirjana Askovic, Jorge Aroche, Mariano Coello, Richard A. Bryant

**Affiliations:** 1 School of Psychology, UNSW Sydney, Sydney, NSW 2052 Australia; 2 Department of Psychiatry, Faculty of Medicine and Health, Northern Clinical School, The University of Sydney, Sydney, NSW Australia; 3 Academic Department of Psychiatry, Royal North Shore Hospital, Northern Sydney Local Health District, St Leonards, Sydney, NSW 2065 Australia; 4 CADE Clinic, Royal North Shore Hospital, Northern Sydney Local Health District, St Leonards, Sydney, NSW 2065 Australia; 5 School of Psychological Sciences, University of Melbourne, Parkville, VIC 3010 Australia; 6 NSW Service for the Treatment and Rehabilitation of Torture and Trauma Survivors (STARTTS), Carramar, NSW 2163 Australia

**Keywords:** Biomarkers, Psychology

Correction to: Translational Psychiatry 10.1038/s41398-022-01795-3, published online 26 January 2022

The original version of this article unfortunately contained a mistake in Figure 2. Unfortunately, the direction of the label is incorrect. Currently it reads “Significant effects of Group: TS – NTS”. The correct title is in fact “Significant Effects of Group: NTS – TS”. All other details in the paper, including the reporting of results and discussion of findings are correct as published. The original article has been corrected.

