# Supplementary material for: Torture exposure and the functional brain: investigating disruptions to intrinsic network connectivity using resting state fMRI
Source: Transl Psychiatry. 2022 Jan 26;12:37. doi: 10.1038/s41398-022-01795-3 (PMC8791936; doi:10.1038/s41398-022-01795-3)
Supplement: Supplementary file 1 — Supplementary Material for paper [file 41398_2022_1795_MOESM1_ESM.docx]

**Supplementary Material**

**Supplementary Methods: fMRI Data Analysis**

Pre-processing of fMRI data

Pre-processing used a combination of toolboxes SPM8 <https://www.fil.ion.ucl.ac.uk/spm/software/spm8/>) and GIFT <https://trendscenter.org/software/gift/>). Each subject’s functional and structural images were first inspected visually for scanner artifacts and gross anatomical abnormalities, and then re-oriented so that the origin of the image lay within 3cm of the anterior commissure. The initial 5 images were discarded to remove longitudinal equilibration effects. A rigid body motion correction was performed using the INRIAlign – a motion correction algorithm ^1^, which is unbiased by local signal changes. This was followed by slice time correction, using the middle slice as the reference frame, to account for timing differences in slice acquisition. Then fMRI data were despiked to mitigate the impacts of outliers using the Despike algorithm implemented within the GIFT software. These images were then spatially normalized to a common stereotactic space using the Montreal Neurological Institute (MNI) EPI template and spatially smoothed with a Gaussian kernel of 8mm^3^ full width at half maximum. Following spatial normalization, the data (originally acquired at 3.75 x 3.75 x 5 mm^3^) were slightly subsampled to 3 x 3 x 3 mm^3^, resulting in 53 x 63 x 46 voxels. Finally, prior to performing group independent component analysis (ICA), each voxel time course was variance normalized as this approach has been shown to yield better decompositions of subcortical and cortical sources ^2^.

Group spatial ICA

Group spatial ICA ^3,4^, as implemented in the GIFT software was used to identify components that exhibited a unique time course profile. From the pre-processed fMRI data of all participants the number of independent “sources”/components/networks were determined using the minimum description length (MDL) criteria ^5^. This number of components were identified using two data reduction steps. The first data reduction step was performed on each subject to reduce the number of time points using principal component analysis (PCA). Then data from all subjects were concatenated and reduced further using PCA and followed by an independent component estimation using the Infomax algorithm ^6^. This algorithm was repeated 10 times in ICASSO (http://research.ics.tkk.fi/ica/icasso/) and the most central run was selected for further analysis. Following group decomposition, single subject time courses (TCs) and spatial maps (SMs) were then back-reconstructed using GICA and calibrated using z scores. Finally, components were visually inspected for artefacts.

Post-ICA processing

Networks for analysis were chosen on the basis of three conditions. First a component’s peak activation clusters should fall on grey matter and it should show low spatial overlap with known vascular, ventricular, susceptibility, and edge regions corresponding to head motion. Second, a component should show more spectral power in the low frequency range (0.01 Hz - 0.10 Hz) compared to the high frequency range (0.15 Hz - 0.25 Hz) ^7^. Finally, in line with our hypotheses, a component should represent either DMN, CEN, SN or any regulatory frontal networks showing primary activity in the lateral and medial PFC.

To remove remaining noise sources including scanner drift and movement related artifacts, the subject specific TCs from the selected components were detrended, and orthogonalized with respect to estimated subject motion parameters. The impact of movement “spikes” on subsequent functional network connectivity (FNC) measures was reduced by despiking the TCs ^2^.

Within and between network functional connectivity

The MANCOVAN toolbox within GIFT software was used to determine *within* network functional connectivity. Within network connectivity was measured using spatial maps (SMs) of networks and also distribution of spectral powers (SPs) at different frequencies. To determine functional connectivity *between* networks, the temporal dynamic functional network connectivity (dFNC) toolbox within the GIFT software was used. Data analyses using dFNC have been conducted in keeping with procedures outlined in previous studies ^2,8^. To compute dFNC between chosen network time courses, a sliding window approach was adopted where the window segment was tapered by convolving a rectangle (width = 20 TRs = 40 s) with a Gaussian (σ = 3 TRs), and advancing 1 TR at each step ^9^, which resulted in W = 128 windows. The chosen window length 40 s (2 s × 20), has been suggested to be suitable for capturing dynamics in FNC ^10^. First, covariance between components was estimated and then the covariance matrices for each window were concatenated to form a Component x Component x Window array to represent the changes in covariance (correlation) between networks (i.e. components) as a function of time.

Using Matlab’s implementation of *k*-means clustering with the squared Euclidean distance, 500 iterations and 150 replications dynamic FNC windows were partitioned into four clusters. The centroids of these clusters can be treated as a small set of prototype connectivity “states” (these can be thought of as average patterns that subjects tend to return to during the course of the experiment)^11,12^. The optimal number of centroid states was estimated using the elbow criterion, defined as the ratio of within cluster to between cluster distances. A *k* of 4 was obtained using this method in a search window of *k* from 2 to 10 ^2^. To examine the structure of dFNC states across groups, we evaluated group-level dFNC states.

**Supplementary Results**

Supplementary Table 1: Torture exposure – event type and duration for the torture survivor group (N = 37)

| **Torture event category (defined under the Istanbul protocol)** | **Experienced and/or witnessed** | **Single event** | **Multiple events** | **Prolonged exposure** |
| --- | --- | --- | --- | --- |
| Deprivation torture (e.g. solitary confinement) | 31 (83.8%) | 1 (3.2%) | 8 (25.8%) | 22 (71.0%) |
| Sensory discomfort (e.g. bright lights) | 22 (59.5%) | 0 | 5 (22.7%) | 17 (77.3%) |
| Forced positioning (e.g. suspension) | 21 (56.8%) | 0 | 8 (38.1%) | 13 (61.9%) |
| Physical torture (e.g. electric shocks) | 30 (81.1%) | 2 (6.7%) | 14 (46.7%) | 14 (46.7%) |
| Humiliating treatment (e.g. verbal abuse) | 26 (70.3%) | 2 (7.7%) | 8 (30.8%) | 16 (61.5%) |
| Psychological torture (e.g. mock executions) | 29 (78.4%) | 2 (6.9%) | 8 (27.6%) | 19 (65.5%) |
| Sexual torture | 9 (24.3%) | 1 (11.1%) | 5 (55.6%) | 3 (33.3%) |

Torture severity index: Computed by counting exposure to torture event category, with experienced as a single event being scored as 1, multiple events scored as 2, and prolonged exposure scored as 3. The minimum score was therefore 1 (1 category experienced once) and the maximum score as 21 (events experienced in all 7 categories at sustained levels).

**Supplementary Table 2: Regions that constitute the 8 ICNS**

| Networks and regions | Brodmann Area | Volume (cc) | t_max_ | MNI (x, y, z) |
| --- | --- | --- | --- | --- |
|  | | | | |
| **Temporo-parietal Default Mode Network (tpDMN)** | | | | |
| Middle Temporal Gyrus | 19, 21, 22, 37, 38, 39 | 16.6/23.0 | 17.9/42.1 | (-56, -36, -2)/(48, -28, -8) |
| Superior Temporal Gyrus | 13, 21, 22, 38, 39, 41, 42 | 17.4/24.4 | 21.0 /38.4 | (-58, -52, 18)/(50, -40, 4) |
| Inferior Parietal Lobule | 40 | 5.5/6.0 | 20.2/25.4 | (-50, -48, 22)/(48, -48, 22) |
| Insula | 13, 22, 40, 41 | 1.5/3.1 | 13.2 /24.7 | (-54, -40, 20)/(42, -28, -4) |
| Supramarginal Gyrus | 40 | 5.4/5.1 | 23.2 /24.6 | (-52, -52, 22)/(52, -52, 22) |
| Inferior Temporal Gyrus | 19, 20, 21, 37 | 0.0/3.6 | 23.9 | (50, -12, -24) |
| Fusiform Gyrus | 19, 20, 37 | 0.1/5.8 | 4.2 /20.6 | (-50, -2, -30)/(50, -8, -28) |
| Parahippocampal Gyrus | 19, 27, 28, 30, 34, 35, 36, 37 | 0.0/9.2 | 18.4 | (40, -36, -16) |
| Inferior Frontal Gyrus | 9, 11, 45, 46, 47 | 5.4/9.9 | 9.2 /15.6 | (-44, 26, -12)/(52, 24, -2) |
| Precuneus | 7, 31 | 4.0/10.7 | 8.9/14.7 | (0, -50, 44)/(8, -52, 44) |
| Middle Occipital Gyrus | 18, 19, 37 | 0.0/7.5 | 13.8 | (42, -68, 2) |
| Middle Frontal Gyrus | 6, 8, 9, 10, 11, 46, 47 | 3.8/10.0 | 7.5 /9.0 | (-42, -2, 56)/(46, 6, 52) |
| **Anterior dorso-medial Default Frontal Network (admDMN)** | | |  |  |
| Medial Frontal Gyrus | 6, 8, 9, 10, 11, 32 | 15.2/16.8 | 55.5 /53.5 | (-2, 54, 18)/(4, 50, 16) |
| Anterior Cingulate | 10, 24, 25, 32 | 8.1/7.7 | 50.0/45.9 | (-2, 48, 10)/(2, 46, 12) |
| Superior Frontal Gyrus | 6, 8, 9, 10 | 22.9/20.2 | 48.8/42.5 | (-10, 52, 36)/(6, 54, 28) |
| Cingulate Gyrus | 23, 24, 31, 32 | 13.4/12.3 | 42.5/35.1 | (-4, -52, 28)/(6, -52, 26) |
| Precuneus | 7, 19, 23, 31, 39 | 8.9/4.9 | 39.3/31.3 | (-4, -52, 32)/(4, -52, 30) |
| Posterior Cingulate | 23, 29, 30, 31 | 4.3/2.9 | 37.1/31.4 | (-4, -54, 24)/(6, -52, 22) |
| Middle Frontal Gyrus | 6, 8, 9, 10, 11, 47 | 14.8/10.2 | 33.7/25.1 | (-20, 34, 48)/(24, 34, 48) |
| Angular Gyrus | 39 | 2.2/0.8 | 30.2 /17.5 | (-48, -64, 36)/(50, -60, 36) |
| Inferior Frontal Gyrus | 9, 10, 13, 44, 45, 46, 47 | 13.2/15.2 | 28.5 /20.2 | (-40, 24, -18)/(30, 20, -20) |
| Inferior Parietal Lobule | 7, 39, 40 | 4.4/1.7 | 27.9 /13.4 | (-50, -62, 40)/(54, -54, 38) |
| Sub-Gyral | 7, 8, 31, 40, 47 | 21.1/24.5 | 26.8/23.9 | (-18, 28, 44)/(12, 38, 4) |
| Supramarginal Gyrus | 40 | 3.6/2.8 | 26.1 /19.9 | (-48, -60, 32)/(50, -58, 32) |
| Superior Temporal Gyrus | 21, 22, 38, 39, 41 | 7.8/4.7 | 22.9/17.6 | (-44, 22, -18)/(38, 20, -22) |
| Inferior Temporal Gyrus | 19, 20, 21, 37 | 2.7/3.2 | 20.8 /14.2 | (-58, -8, -26)/(62, -8, -26) |
| Middle Temporal Gyrus | 19, 21, 37, 39 | 16.5/10.8 | 20.2/12.9 | (-48, -66, 28)/(46, 8, -40) |
| **Anterior ventro-medial Default Frontal Network (admDMN)** | | |  |  |
| Sub-Gyral | 4, 6, 8, 20, 31, 37, 40 | 52.8/46.3 | 39.7/35.1 | (-16, 20, -10)/(22, 40, 0) |
| Extra-Nuclear | 13, 47 | 12.4/12.7 | 39.0/29.6 | (-10, 14, -10)/(16, 30, 2) |
| Caudate | * | 2.9/2.9 | 36.1 /32.2 | (-8, 14, -6)/(6, 12, -6) |
| Anterior Cingulate | 9, 10, 24, 25, 32, 33 | 7.1/7.6 | 34.5 )/30.8 | (-6, 14, -10)/(16, 46, -2) |
| Middle Frontal Gyrus | 6, 8, 9, 10, 11, 46, 47 | 13.8/14.1 | 30.6)/31.8 | (-24, 44, -2)/(26, 40, -2) |
| Medial Frontal Gyrus | 6, 8, 9, 10, 11, 32 | 11.1/11.7 | 28.8/30.9 | (-20, 44, -4)/(20, 46, -4) |
| Inferior Frontal Gyrus | 9, 10, 11, 44, 45, 46, 47 | 10.4/9.1 | 28.2 /23.2 | (-22, 28, -10)/(36, 40, 2) |
| Superior Frontal Gyrus | 6, 8, 9, 10 | 6.1/7.0 | 27.8/24.9 | (-18, 50, 4)/(22, 50, 4) |
| **Posterior dorso-medial Default Mode Network (pdmDMN)** | | | | |
| Precuneus | 7, 18, 19, 23, 31, 39 | 25.9/25.4 | 46.6/ | (-12, -62, 20)/(6, -60, 38) |
| Posterior Cingulate | 23, 29, 30, 31 | 5.2/5.0 | 45.0 /45.2 | (-8, -60, 24)/(8, -60, 22) |
| Cingulate Gyrus | 23, 31 | 8.0/7.4 | 42.9 /44.5 | (-4, -62, 26)/(8, -60, 26) |
| Sub-Gyral | 6, 7, 31, 40 | 14.5/18.9 | 38.8/41.6 | (-12, -58, 24)/(16, -56, 24) |
| Middle Temporal Gyrus | 19, 21, 22, 37, 39 | 10.0/13.1 | 29.7 /39.7 | (-42, -72, 26)/(44, -64, 24) |
| Angular Gyrus | 39 | 2.0/1.5 | 31.5/38.5 | (-42, -72, 30)/(40, -70, 32) |
| Cuneus | 7, 18, 19, 30 | 6.2/4.7 | 35.5 /37.6 | (-10, -70, 30)/(8, -68, 32) |
| Superior Temporal Gyrus | 13, 22, 39, 41, 42 | 6.4/7.9 | 21.6/33.7 | (-46, -60, 26)/(48, -62, 26) |
| Superior Parietal Lobule | 5, 7, 40 | 4.4/5.2 | 29.4/25.9 | (-8, -70, 56)/(12, -68, 56) |
| Inferior Parietal Lobule | 7, 39, 40 | 4.5/9.2 | 27.2/26.9 | (-36, -68, 40)/(38, -68, 40) |
| Supramarginal Gyrus | 40 | 3.4/3.6 | 19.8/25.9 | (-46, -56, 26)/(48, -54, 22) |
| Medial Frontal Gyrus | 6, 9, 10, 11, 32 | 5.1/6.8 | 21.8/20.9 | (-2, 50, -8)/(4, 54, -8) |
| **Left Central Executive Network (lCEN)** | | | | |
| Middle Frontal Gyrus | 6, 8, 9, 10, 11, 46, 47 | 31.0/10.3 | 40.7/14.1 | (-42, 40, -6)/(36, 40, -12) |
| Inferior Parietal Lobule | 7, 39, 40 | 14.5/3.0 | 39.7/16.4 | (-36, -66, 44)/(40, -62, 46) |
| Superior Parietal Lobule | 7, 40 | 4.5/1.4 | 36.2/14.6 | (-36, -70, 46)/(40, -62, 50) |
| Inferior Frontal Gyrus | 9, 10, 13, 44, 45, 46, 47 | 21.4/3.8 | 35.9/11.8 | (-48, 20, 24)/(36, 36, -10) |
| Precuneus | 7, 19, 23, 31, 39 | 11.8/1.2 | 34.9/8.5 | (-36, -70, 42)/(2, -66, 34) |
| Superior Frontal Gyrus | 6, 8, 9, 10 | 19.6/1.8 | 32.3/8.7 | (-32, 12, 56)/(2, 30, 50) |
| Sub-Gyral | 6, 8, 32, 37 | 31.5/5.2 | 31.5/8.0 | (-32, -64, 42)/(12, -28, 64) |
| Middle Temporal Gyrus | 19, 21, 22, 37, 39 | 19.5/3.6 | 29.8 /9.1 | (-66, -40, -6)/(64, -38, -6) |
| Angular Gyrus | 39 | 2.3/1.0 | 28.8/10.4 | (-48, -66, 34)/(44, -64, 34) |
| Precentral Gyrus | 6, 9, 43, 44 | 20.2/5.1 | 28.2/10.7 | (-44, 20, 38)/(12, -28, 68) |
| Supramarginal Gyrus | 40 | 5.0/0.3 | 23.7/7.4 | (-48, -60, 30)/(44, -56, 36) |
| Superior Temporal Gyrus | 13, 21, 22, 38, 39, 41, 42 | 15.0/5.5 | 21.8/7.4 | (-54, -64, 28)/(58, -26, 0) |
| Declive | * | 0.6/8.6 | 5.1/20.0 | (-10, -82, -26)/(14, -80, -28) |
| Cingulate Gyrus | 23, 24, 31, 32 | 8.0/2.6 | 19.2/15.9 | (-2, -34, 36)/(2, -34, 36) |
| Inferior Temporal Gyrus | 19, 20, 21, 37 | 3.0/0.0 | 19.1 | (-60, -56, -10) |
| Medial Frontal Gyrus | 6, 8, 9, 10, 11, 32 | 11.5/2.3 | 19.1/11.1 | (-6, 26, 52)/(2, 46, -18) |
| **Right Central Executive Network (rCEN)** | | | | |
| Inferior Parietal Lobule | 7, 39, 40 | 10.2/11.7 | 24.4 (-40, -52, 47)/47.8 | (-40, -56, 48)/(44, -54, 44) |
| Angular Gyrus | 39 | 1.2/1.3 | 11.6 (-36, -55, 36)/36.9 | (-36, -58, 36)/(42, -58, 36) |
| Middle Frontal Gyrus | 6, 8, 9, 10, 11, 46, 47 | 13.6/31.0 | 13.3 (-38, 44, -4)/36.3 | (-38, 46, -2)/(42, 24, 44) |
| Supramarginal Gyrus | 40 | 1.1/4.2 | 13.1 (-40, -49, 37)/35.4 | (-40, -52, 38)/(44, -54, 36) |
| Superior Frontal Gyrus | 6, 8, 9, 10 | 5.0/21.6 | 11.1 (-28, 14, 51)/34.1 | (-28, 12, 56)/(36, 16, 54) |
| Superior Parietal Lobule | 7, 40 | 3.6/3.4 | 19.8 (-40, -56, 51)/32.1 | (-40, -60, 52)/(40, -60, 52) |
| Precentral Gyrus | 4, 6, 9, 13, 43, 44 | 7.0/8.3 | 10.4 (-34, -20, 56)/30.6 | (-34, -24, 60)/(44, 22, 40) |
| Inferior Frontal Gyrus | 9, 10, 13, 44, 45, 46, 47 | 0.9/17.5 | 12.3 (-38, 45, 0)/27.3 | (-38, 46, 2)/(40, 54, 4) |
| Superior Temporal Gyrus | 13, 21, 22, 38, 39, 41, 42 | 6.0/12.2 | 8.8 (-44, -23, 9)/25.7 | (-44, -24, 8)/(48, -58, 28) |
| Medial Frontal Gyrus | 6, 8, 9, 10, 11, 32 | 2.4/15.3 | 12.1 (-2, 29, 41)/23.9 | (-2, 28, 46)/(8, 26, 50) |
| Sub-Gyral | 6, 10, 30 | 12.2/19.7 | 14.3 (-28, -80, -1)/23.6 | (-28, -82, -6)/(34, -58, 42) |
| Cingulate Gyrus | 23, 24, 31, 32 | 3.7/10.2 | 18.8 (0, -29, 33)/22.6 | (0, -32, 34)/(6, -36, 36) |
| Postcentral Gyrus | 1, 2, 3, 5, 40, 43 | 5.4/5.8 | 11.4 (-42, -26, 55)/19.4 | (-42, -30, 58)/(54, -38, 54) |
| Declive | * | 10.2/0.0 | 19.3 | (-12, -80, -26) |
| Precuneus | 7, 19, 31, 39 | 4.9/11.4 | 13.9)/18.7 | (-30, -64, 40)/(38, -66, 36) |
| Middle Temporal Gyrus | 20, 21, 22, 37, 39 | 2.4/10.1 | 8.8/18.5 | (-60, -46, -12)/(66, -34, -12) |
| **lateral Frontal Network (latFN)** | | |  |  |
| Inferior Frontal Gyrus | 9, 10, 11, 13, 44, 45, 46, 47 | 21.6/23.2 | 34.8/50.4 | (-42, 30, -2)/(52, 32, 14) |
| Middle Frontal Gyrus | 6, 8, 9, 10, 11, 46, 47 | 12.5/21.7 | 28.0/43.8 | (-44, 34, -4)/(52, 32, 18) |
| Sub-Gyral | 6, 7, 21, 37, 39, 40, 47 | 14.3/27.8 | 23.9/35.6 | (-44, 18, 20)/(48, 14, 18) |
| Precentral Gyrus | 3, 6, 9, 43, 44 | 3.1/14.1 | 26.9/34.9 | (-50, 16, 10)/(50, 18, 10) |
| Insula | 13 | 5.6/9.8 | 27.9/30.4 | (-38, 20, 2)/(46, 10, 14) |
| Extra-Nuclear | 13 | 5.2/11.6 | 29.2/17.6 | (-34, 22, 0)/(40, 14, -10) |
| Superior Temporal Gyrus | 13, 22, 38, 39, 41, 42 | 9.5/13.4 | 18.5/23.1 | (-46, 16, -12)/(52, 18, -8) |
| Postcentral Gyrus | 1, 2, 3, 5, 40, 43 | 0.3/11.1 | 6.1/19.7 | (-56, -32, 40)/(60, -30, 48) |
| Inferior Parietal Lobule | 40 | 4.2/12.1 | 9.2/18.1 | (-58, -40, 32)/(60, -34, 46) |
| Middle Temporal Gyrus | 19, 21, 22, 37, 39 | 5.4/10.3 | 11.0 /15.9 | (-48, -56, -4)/(36, -74, 26) |
| Caudate | * | 1.6/2.7 | 10.3/15.7 | (-10, 10, 10)/(12, 8, 8) |
| Supramarginal Gyrus | 40 | 1.6/2.4 | 9.7 /15.2 | (-56, -40, 36)/(56, -40, 36) |
| Superior Frontal Gyrus | 6, 8, 9, 10 | 4.4/8.4 | 10.9/14.5 | (-2, 30, 50)/(6, 20, 56) |
| Precuneus | 7, 19, 31 | 0.6/7.2 | 6.2 /13.7 | (-24, -80, 34)/(28, -70, 34) |
| **dorsal Frontal Network (dorFN)** | | |  |  |
| Superior Frontal Gyrus | 6, 8, 9, 10 | 18.5/21.6 | 39.1/43.7 | (-22, 8, 58)/(24, 6, 58) |
| Sub-Gyral | 6, 10, 21, 32 | 24.6/32.8 | 32.8 /40.9 | (-24, 4, 56)/(20, 6, 58) |
| Middle Frontal Gyrus | 6, 8, 9, 10, 46 | 23.3/26.1 | 33.4/39.6 | (-22, 18, 52)/(28, 8, 58) |
| Medial Frontal Gyrus | 6, 8, 9, 32 | 11.3/14.9 | 33.2/36.7 | (-18, 8, 54)/(18, 8, 52) |
| Cingulate Gyrus | 24, 31, 32 | 10.4/12.7 | 26.9/33.8 | (-18, 6, 50)/(20, 4, 50) |
| Precentral Gyrus | 4, 6, 9, 44 | 9.9/13.2 | 23.3/28.8 | (-32, -8, 56)/(32, -8, 54) |
| * | * | 4.7/4.1 | 14.9/20.1 | (-18, 10, 70)/(2, 22, 60) |
| Inferior Frontal Gyrus | 6, 9, 44, 47 | 4.2/2.9 | 13.6/15.4 | (-36, 6, 32)/(52, 8, 34) |
| Precuneus | 7, 18, 19, 23, 31, 39 | 6.5/4.4 | 15.0/12.1 | (-36, -76, 34)/(36, -78, 36) |
| Angular Gyrus | 39 | 1.1/1.0 | 13.8/11.6 | (-36, -76, 30)/(38, -78, 32) |
| Posterior Cingulate | 23, 29, 30, 31 | 3.3/4.3 | 13.6/12.1 | (-16, -60, 14)/(18, -58, 14) |
| Middle Temporal Gyrus | 19, 20, 21, 37, 38, 39 | 8.8/11.8 | 12.2/13.5 | (-36, -72, 28)/(42, -68, 20) |
| Extra-Nuclear | * | 4.5/5.3 | 10.4/12.5 | (-12, -60, 18)/(14, -58, 16) |
| Anterior Cingulate | 32 | 1.1/1.4 | 9.3/11.3 | (-6, 8, 26)/(22, 34, 28) |
| Superior Temporal Gyrus | 13, 21, 22, 38, 39, 41 | 2.6/7.9 | 7.5/11.1 | (-50, -10, 0)/(46, -62, 16) |

**Supplementary Table 3**: Means and standard deviations of degree of internetwork connectivity in torture survivor and non-torture survivor groups.

|  | **Torture survivor (N = 37)** | | **Non-torture survivor (N = 62)** | |
| --- | --- | --- | --- | --- |
| Internetwork Connectivity | Mean | SD | Mean | SD |
| lCEN – admDMN | 0.213 | 0.24 | 0.174 | 0.21 |
| lCEN – avmDMN | 0.058 | 0.12 | 0.026 | 0.08 |
| latFr – lCEN | -0.131 | 0.15 | -0.136 | 0.17 |
| LatFr - admDMN | -0.173 | 0.21 | -0.136 | 0.17 |

**Supplementary Figure 1:** 25 networks identified by independent components analysis. The 8 networks that aligned with hypothesized networks of interest are labelled as follows: 5: tpDMN – temporparietal default mode network; 10: admDMN - anterior dorsomedial default mode network; 15: avmDMN – anterior ventromedial default mode network; 16: lCEN – left central executive network; 17: latFN – lateral frontal network; 18: dorFN – dorsal frontal network; 20: rCEN – right central executive network; 21: pdDMN – posterior dorsal default mode network.

**
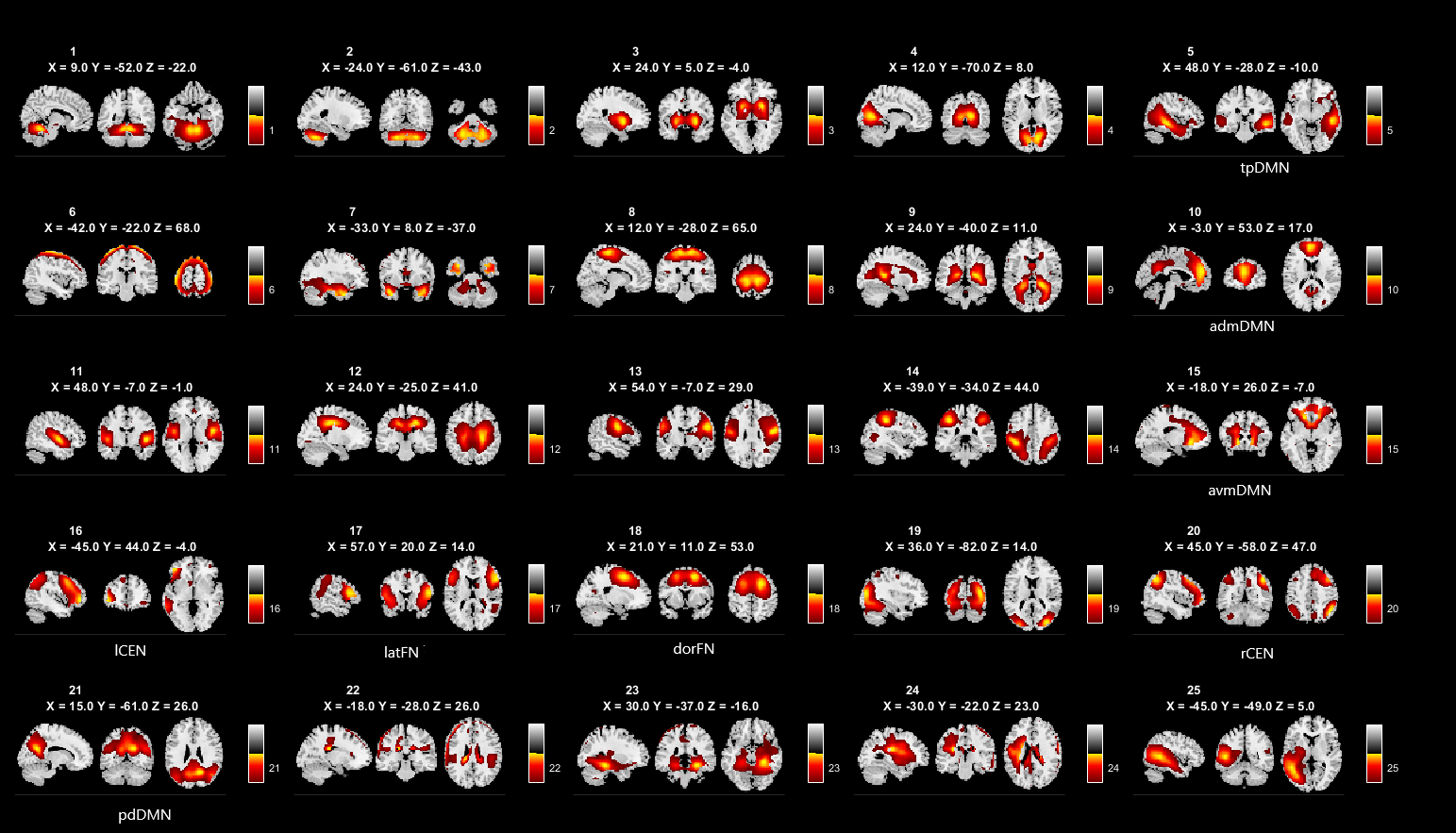
**

**References**

1 Freire, L. & Mangin, J. F. Motion correction algorithms may create spurious brain activations in the absence of subject motion. *Neuroimage.* **14**, 709-722. (2001).

2 Damaraju, E. *et al.* Dynamic functional connectivity analysis reveals transient states of dysconnectivity in schizophrenia. *Neuroimage Clin.* **5:298-308.**, 10.1016/j.nicl.2014.1007.1003. eCollection 2014. (2014).

3 Calhoun, V. D., Adali, T., Pearlson, G. D. & Pekar, J. J. A method for making group inferences from functional MRI data using independent component analysis. *Human Brain Mapping* **14**, 140-151 (2001).

4 Erhardt, E. B. *et al.* Comparison of multi-subject ICA methods for analysis of fMRI data. *Human brain mapping* **32**, 2075-2095, doi:10.1002/hbm.21170 (2011).

5 Li, Y. O., Adali, T. & Calhoun, V. D. Estimating the number of independent components for functional magnetic resonance imaging data. *Human brain mapping* **28**, 1251-1266 (2007).

6 Bell, A. J. & Sejnowski, T. J. An information-maximization approach to blind separation and blind deconvolution. *Neural computation* **7**, 1129-1159 (1995).

7 Allen, E. A. *et al.* A baseline for the multivariate comparison of resting-state networks. *Front Syst Neurosci.* **5:2.**, 10.3389/fnsys.2011.00002. eCollection 02011. (2011).

8 Malhi, G. S., Das, P., Outhred, T., Bryant, R. A. & Calhoun, V. Resting-state neural network disturbances that underpin the emergence of emotional symptoms in adolescent girls: resting-state fMRI study. *The British journal of psychiatry : the journal of mental science* **215**, 545-551, doi:10.1192/bjp.2019.10 (2019).

9 Du, Y. *et al.* Interaction among subsystems within default mode network diminished in schizophrenia patients: a dynamic connectivity approach. *Schizophrenia research* **170**, 55-65, doi:10.1016/j.schres.2015.11.021 (2016).

10 Du, Y. *et al.* Dynamic functional connectivity impairments in early schizophrenia and clinical high-risk for psychosis. *NeuroImage*, doi:<https://doi.org/10.1016/j.neuroimage.2017.10.022> (2017).

11 Calhoun, V. D., Miller, R., Pearlson, G. & Adali, T. The chronnectome: time-varying connectivity networks as the next frontier in fMRI data discovery. *Neuron.* **84**, 262-274. doi: 210.1016/j.neuron.2014.1010.1015. Epub 2014 Oct 1022. (2014).

12 Miller, R. L. *et al.* Higher Dimensional Meta-State Analysis Reveals Reduced Resting fMRI Connectivity Dynamism in Schizophrenia Patients. *PLoS One.* **11**, e0149849. doi: 0149810.0141371/journal.pone.0149849. eCollection 0142016. (2016).
